# Supplementary material for: Efficiently accelerated free electrons by metallic laser accelerator
Source: Nat Commun. 2023 Sep 20;14:5857. doi: 10.1038/s41467-023-41624-9 (PMC10511530; doi:10.1038/s41467-023-41624-9)
Supplement: Supplementary file 1 — Supplementary Information [file 41467_2023_41624_MOESM1_ESM.pdf]

Supplementary Information

Efficiently accelerated free electrons by metallic laser accelerator

Dingguo Zheng[1,2], Siyuan Huang[1,2], Jun Li[1], Yuan Tian[1,2], Yongzhao Zhang[1,2], Zhongwen Li[1], Huanfang Tian[1], Huaixin Yang[1,2,3] & Jianqi Li *[1,2,3,4]

[1] Beijing National Laboratory for Condensed Matter Physics, Institute of Physics, Chinese Academy of Sciences, Beijing, 100190, China.

[2] School of Physical Sciences, University of Chinese Academy of Sciences, Beijing, 100049, China.

[3] Songshan Lake Materials Laboratory, Dongguan, Guangdong, 523808, China.

[4] Department of Electrical and Electronic Engineering, Southern University of Science and Technology, Shenzhen, Guangdong, 518055, China.

*Corresponding author: ljq@iphy.ac.cn

Table of content:

Supplementary note 1: Evanescent wave and accelerating gradient of MLA

Supplementary note 2: Interaction of electron with multimode field

Supplementary Figures

Supplementary References

Supplementary Note 1: Evanescent wave and accelerating gradient of MLA

Because of the response of free electrons in metal, the incident light could be efficiently reflected by a flat metallic surface. For the sake of simplicity, we consider the case that the reflectivity equals to 1. The interaction of incident light with reflected light forms standing wave, which could be expressed as

$$E_0 e^{-\frac{y^2+z^2}{2\sigma}} e^{\mathrm{i}(k_0 x-\omega t)} - E_0 e^{-\frac{y^2+z^2}{2\sigma}} e^{\mathrm{i}(-k_0 x-\omega t)} = 2\mathrm{i}E_0 e^{-\frac{y^2+z^2}{2\sigma}} \sin k_0 x\, e^{-\mathrm{i}\omega t}, \quad (1)$$

where E_0 is the amplitude of incident light, k_0 is the wavevector in vacuum, ω is the angular frequency, σ is the standard deviation in y-z plane. The amplitude of electric field $2E_0 e^{-\frac{y^2+z^2}{2\sigma}} \sin k_0 x$ is independent of time. If a free electron with velocity v_e goes through the field along the z-axis direction, it experiences the electric field of $2\mathrm{i}E_0 e^{-\frac{y^2+z^2}{2\sigma}} \sin k_0 x\, e^{-\mathrm{i}\omega\frac{z}{v_\mathrm{e}}}$ at $t = \frac{z}{v_\mathrm{e}}$. Thus, the energy change of the electron after it goes through the field is

$$\Delta E_\mathrm{e} = e\int_{-\infty}^{+\infty} 2\mathrm{i}E_0 e^{-\frac{y^2+z^2}{2\sigma}} \sin k_0 x\, e^{-\mathrm{i}\omega\frac{z}{v_\mathrm{e}}}\, \mathrm{d}z = 0, \quad (2)$$

when $\sigma \gg \frac{2\pi}{k_0}$. This result demonstrates that the electron energy cannot be affected by these standing waves. However, nearby the grating surface, the incident light can be tailored by the periodic nanostructure of MLA, resulting in a periodical electric field along the electron propagating direction. The expression of the evanescent wave should contain a term $U(z)$, which satisfies $U(z+\Lambda) = U(z)$ with Λ being the grating spatial period[1,2]. The Fourier series expansion of $U(z)$ could be expressed as

$$U(z) = \sum_n a_n e^{\mathrm{i}n\frac{2\pi}{\Lambda}z}, \quad (3)$$

where n is a positive integer, a_n is the nth Fourier coefficient.

Assuming the grating is infinite along the y-direction and the light polarization is along the z-direction, we can obtain the excited transverse magnetic wave with three components, electric field E_x, E_z and magnetic field B_y. E_z can be expressed as

$$E_z(x,z,t) = E_0 U(z) e^{i(k_x x - \omega t)} = E_0 \sum_n a_n \, e^{\mathrm{i}(k_z z + k_x x - \omega t)}, \tag{4}$$

where $k_z = n\frac{2\pi}{\Lambda}$ and k_x is the component of wavevector along the z and x-direction, respectively. The phase-matching between the electron propagation and the periodic electric field requires that $\Lambda = \beta\lambda$, in which $\beta = \frac{v_\mathrm{e}}{c}$ and c is light speed in vacuum. Hence, k_z can also be written as $k_z = n\frac{k_0}{\beta}$. using the wave equation $\nabla^2\mathbf{E} - \frac{1}{c^2}\frac{\partial^2}{\partial t^2}\mathbf{E} = 0$, we can obtain that $k_x = \mathrm{i}\frac{k_0}{\beta}\sqrt{n^2-\beta^2}$. then we can define $\delta_n = \frac{\beta}{k_0\sqrt{n^2-\beta^2}}$ as the decay length of evanescent field along the x-direction, so equation (4) can be rewritten as

$$E_z(x,z,t) = E_0 \sum_n a_n e^{\mathrm{i}\left(n\frac{k_0}{\beta}z - \omega t\right)} e^{-\frac{x}{\delta_n}}. \tag{5}$$

By solving Maxwell equations $\nabla\cdot\mathbf{E} = 0$ and $\nabla\times\mathbf{E} = -\frac{\partial\mathbf{B}}{\partial t}$, we can obtain the other two components E_x and B_y.

At $t = \frac{z}{v_\mathrm{e}} = \frac{z}{\beta c}$, the electron can be accelerated or decelerated which depends on the initial phase ϕ_0 of the evanescent wave. The electron experienced electric field can be expressed as

$$E_z(x,z) = E_0 e^{\mathrm{i}\phi_0} \sum_n a_n e^{\mathrm{i}\frac{k_0}{\beta}(n-1)z} e^{-\frac{x}{\delta_n}}. \tag{6}$$

Considering the electron energy variation $\Delta E = E_\mathrm{e}$ is much small than the initial electron energy $E_{\mathrm{e},0}$, then ΔE_e can be expressed as

$$\Delta E_\mathrm{e} = eN\int_0^\Lambda E_z(x,z)dz = eNE_0 e^{\mathrm{i}\phi_0}\left(\Lambda a_1 e^{-\frac{x}{\delta_1}} + \sum_{n=1} a_{n+1} e^{-\frac{x}{\delta_{n+1}}} \int_0^\Lambda e^{\mathrm{i}n\frac{k_0}{\beta}z} dz\right), \tag{7}$$

where N is the number of spatial periods of MLA, $\delta_1 = \frac{\beta}{k_0\sqrt{1-\beta^2}} = \frac{\beta\gamma}{k_0}$. The integral result is zero in the second term, which indicated that all the high-order terms do not contribute to the energy changes, thus

$$\Delta E_\mathrm{e} = eN\Lambda E_0 a_1 e^{-\frac{x}{\delta_1}} e^{\mathrm{i}\phi_0}. \tag{8}$$

Here the coefficient a_1 is related to the grating material and nanostructural features. According the theoretical analysis of DLA, the acceleration gradient G can be defined as the effective electric field over one period, yielding[1,2]

$$G = \frac{\Delta E_{\mathrm{e}}}{N\Lambda} = eE_0 a_1 e^{\mathrm{i}\phi_0} e^{-\frac{x}{\delta_1}}. \tag{9}$$

Supplementary Note 2: Interaction of electron with multimode field

In conventional PINEM theoretical studies[3,4], the electron wavefunction and its quantized energy eigenvalues have been investigated. Recently, quantum optics theory has also been introduced to solve the interaction of electron with cavity photon[5,6]. In this paper, we focus on the interaction of electron with the multimode field, we firstly start by solving the time-dependent Schrödinger equation.

In our experiments, the electron transverse momentum is far below its longitudinal momentum, we can safely ignore the transverse movements during the interaction in the following discussion.

2.1. Electron-photon interaction strength

The interaction Hamiltonian for a single electron with an electromagnetic field can be written as

$$\hat{H} = \frac{1}{2m_{\mathrm{e}}}(\hat{p} - e\mathbf{A})^2 + e\mathrm{V}, \tag{10}$$

where m_{e} is the electron mass, e is electron charge, $\mathbf{A}$ is the vector potential, and V is the scalar potential. In the absence of extra charges, $\mathrm{V} = 0$. The pondermotive term $(\frac{e^2}{2m_{\mathrm{e}}}\mathbf{A}^2)$ related with Kapitza-Dirac effect is small under the experimental conditions of the study and can be neglected. The Hamiltonian can be rearranged as $\hat{H} = \hat{H}_0 + \hat{H}_1$, where the ground state Hamiltonian

$$\hat{H}_0 = \frac{1}{2m_{\mathrm{e}}}\hat{p}^2 \approx E_{\mathrm{e},0} + \mathbf{v}_{\mathrm{e}}(\hat{p} - \mathbf{p}_0) \tag{11}$$

and the interaction term

$$\hat{H}_1 = -\frac{e}{m_{\mathrm{e}}}\mathbf{A} \cdot \hat{p}, \tag{12}$$

using the Coulomb gauge $\nabla \cdot \mathbf{A} = 0$.

Considering the interaction along the z-direction, $\hat{p} = -\mathrm{i}\hbar\frac{\partial}{\partial z}$, we write the initial electron wave function as $\Psi(z,t) = e^{\mathrm{i}(p_0 z - E_{\mathrm{e},0}t)/\hbar}\psi(z,t)$, where $\psi(z,t)$ is the initial envelope function. Using the Schrödinger equation $\mathrm{i}\hbar\partial\Psi(z,t)/\partial t = (\widehat{H}_0 + \widehat{H}_1)\Psi(z,t)$, we obtain

$$\left(\frac{\partial}{\partial t} + v_\mathrm{e}\frac{\partial}{\partial z}\right)\psi(z,t) = -\frac{\mathrm{i}ev_\mathrm{e}}{\hbar}\mathrm{A}_z\psi(z,t), \tag{13}$$

The solution of this equation is

$$\psi(z,t) = \psi_0(z - v_\mathrm{e}t)\phi(z,t) \tag{14}$$

and

$$\phi(z,t) = \exp\left(-\frac{\mathrm{i}ev_\mathrm{e}}{\hbar}\int_{-\infty}^{t}\mathrm{d}t'\mathrm{A}_z\,(z - v_\mathrm{e}t + v_\mathrm{e}t', t')\right), \tag{15}$$

The argument $z - v_\mathrm{e}t + v_\mathrm{e}t'$ is related with the electron propagation. In the multimode fields, the vector potential is the sum of all the modes j,

$$\mathrm{A}_z(z,t) = -\frac{\mathrm{i}}{2\omega}\sum_j E_z^{(j)}(z,t)e^{-\mathrm{i}j\omega t} + \mathrm{c.c.} = -\frac{1}{\omega}\,\mathrm{Im}\left(\sum_j E_z^{(j)}(z,t)e^{-\mathrm{i}j\omega t}\right). \tag{16}$$

Putting expression (16) into equation (15), we have

$$\phi(z,t) = \exp\left(\frac{\mathrm{i}ev_\mathrm{e}}{\hbar\omega}\mathrm{Im}\left(\sum_j\int_{-\infty}^{t}\mathrm{d}t'\,E_z^{(j)}(z - v_\mathrm{e}t + v_\mathrm{e}t', t')e^{-\mathrm{i}j\omega t}\right)\right). \tag{17}$$

Let $z' = z - v_\mathrm{e}t + v_\mathrm{e}t'$, then $t' = \frac{z'}{v_\mathrm{e}} - (\frac{z}{v_\mathrm{e}} - t)$. By converting the temporal integral into special integral, the above equation can be rewritten as

$$\phi(z,t) = \exp\left(\frac{\mathrm{i}e}{\hbar\omega}\mathrm{Im}\left(\sum_j\int_{-\infty}^{z}\mathrm{d}z'\,E_z^{(j)}\left(z', \frac{z'}{v_\mathrm{e}} - \left(\frac{z}{v_\mathrm{e}} - t\right)\right)e^{-\frac{\mathrm{i}j\omega}{v_\mathrm{e}}z'}\,\mathrm{e}^{ij\left(\frac{\omega}{v_\mathrm{e}}z - \omega t\right)}\right)\right). \tag{18}$$

Define the electron-photon interaction strength

$$g_j(z,t) = \frac{e}{2\hbar\omega}\int_{-\infty}^{z}\mathrm{d}z' E_z^{(j)}\left(z', \frac{z'}{v_\mathrm{e}} - \left(\frac{z}{v_\mathrm{e}} - t\right)\right)e^{-\frac{\mathrm{i}j\omega}{v_\mathrm{e}}z'}. \tag{19}$$

If we replace $\frac{z}{v_\mathrm{e}} - t$ with T, the extended PINEM theory can be obtained[7].

For MLA, if only the fundamental near field is excited, $g = g_1$, then the expression of g can be obtained by taking the electric field distribution function (5) into equation

(19). Because the equation (16) contains the time factor $e^{-\mathrm{i}\omega t}$, it should not appear anymore. Hence, the electric field can be expressed as $E_z(x,z) = E_0 \sum_n a_n e^{\mathrm{i}n\frac{k_0}{\beta}z} e^{-\frac{x}{\delta_n}}$, which is time-independent. If the electric field only exists within the range of $0 < z < N\Lambda$ (N is the number of spatial periods of MLA), we can get interaction strength,

$$g = \frac{e}{2\hbar\omega}\int_0^{N\Lambda} \mathrm{d}z\, E_0 \sum_n a_n e^{\mathrm{i}n\frac{k_0}{\beta}z} e^{-\frac{x}{\delta_n}} e^{-\frac{\mathrm{i}\omega}{v_\mathrm{e}}z} = \frac{eN\Lambda}{2\hbar\omega} E_0 a_1 e^{-\frac{x}{\delta_1}}. \tag{20}$$

In comparison with equation (9), the relationship between the PINEM theory parameter g and DLA parameter G is

$$|g| = \frac{N\Lambda}{2\hbar\omega}|G|. \tag{21}$$

2.2. Electron wavefunction in multimode fields

Using the equation $\mathrm{Im}(g_j) = \mathrm{Im}\left(|g_j| e^{\mathrm{i}\arg(g_j)}\right) = |g_j| \sin(\arg(g_j))$, equation (18) can be expressed as

$$\phi(z,t) = \prod_j \phi_j(z,t), \tag{22}$$

where

$$\phi_j(z,t) = \exp\left(2\mathrm{i}|g_j(z,t)| \sin\left(\arg(g_j) + \frac{j\omega}{v_\mathrm{e}}z - j\omega t\right)\right). \tag{23}$$

Using the Jacobi-Anger expansion, $e^{\mathrm{i}x\sin\theta} = \sum_{n=-\infty}^{\infty} J_n(x) e^{\mathrm{i}n\theta}$, we have

$$\phi_j(z,t) = \sum_{n=-\infty}^{\infty} J_n(2|g_j(z,t)|) \exp\left(\mathrm{i}n \arg(g_j) + \mathrm{i}jn\left(\frac{\omega}{v_\mathrm{e}}z - \omega t\right)\right). \tag{24}$$

If only the fundamental and SH field exist,

$$\Psi(z,t) = e^{\frac{\mathrm{i}(p_0 z - E_{\mathrm{e},0}t)}{\hbar}} \psi_0(z - v_\mathrm{e}t)\phi_1(z,t)\phi_2(z,t), \tag{25}$$

where,

$$\phi_1(z,t)\phi_2(z,t) = \sum_{l=-\infty}^{\infty}\sum_{m=-\infty}^{\infty} J_m(2|g_1|)J_l(2|g_2|) \exp\left(\mathrm{i}m \arg(g_1) + \mathrm{i}l \arg(g_2) + \mathrm{i}(m+2l)\left(\frac{\omega}{v_\mathrm{e}}z - \omega t\right)\right). \tag{26}$$

Let $n = m + 2l$, then

$$\phi_1(z,t)\phi_2(z,t) = \sum_{n=-\infty}^{\infty} f_n \exp\left(in\left(\frac{\omega}{v_{\mathrm{e}}}z - \omega t\right)\right), \tag{27}$$

where,

$$f_n = \exp(in\arg(g_1))\sum_{l=-\infty}^{\infty} J_{n-2l}(2|g_1|)J_l(2|g_2|)\exp(-\mathrm{i}l\delta) \tag{28}$$

and

$$\delta = 2\arg(g_1) - \arg(g_2). \tag{29}$$

Then the electron wavefunction can be expressed in the form for a single mode field

$$\Psi(z,t) = \psi_0(z - v_{\mathrm{e}}t)\sum_{n=-\infty}^{\infty} f_n \exp\frac{\mathrm{i}}{\hbar}\left((p_0 + n\frac{\hbar\omega}{v_{\mathrm{e}}})z - (E_{\mathrm{e},0} + n\hbar\omega)t\right). \tag{30}$$

This fact indicates that when an electron's energy increases by $\hbar\omega$ after absorbing a photon, its momentum will increase by $\hbar\omega/v_{\mathrm{e}}$ accordingly. The probability of an electron gaining $n\hbar\omega$ is

$$\mathrm{P}_n(|g_1|,|g_2|,\delta) = |f_n|^2 = \sum_{l=-\infty}^{\infty}\sum_{m=-\infty}^{\infty} \alpha_{lm}\exp(-\mathrm{i}(l-m)\delta), \tag{31}$$

where

$$\alpha_{lm} = J_{n-2m}(2|g_1|)J_{n-2l}(2|g_1|)J_m(2|g_2|)J_l(2|g_2|). \tag{32}$$

Here, the probability not only depends on the amplitude of the near field, but also on the relative phase of the fundamental near field and the SH near field.

2.3. Electron energy spectrum under nonuniform near field

For free electrons having initial energy distribution (zero-loss peak) $\rho_i(E_0)$, after interacting with the near field, the final electron energy distribution can be expressed as

$$\rho_f(E) = \sum_n \mathrm{P}_n\rho_i(E_{\mathrm{e},0} + n\hbar\omega). \tag{33}$$

However, in most experiments, the duration of the pump laser pulse is shorter than that of the electron pulse, except stretching pump laser pulse. Furthermore, the near field is rarely uniform for an electron beam with a spot size of tens micrometer. Therefore, the electrons in a pulse would experience different interaction strengths. Previous

investigations don't consider the alterations of interaction strengths at different time delays. We can use another distribution function $\mathrm{D}(|g|)$ to illustrate the probability of an electron interacting with the near field $|g|$, which satisfies $\int \mathrm{D}(|g|)\mathrm{d}|g| = 1$. Therefore, the electron energy distribution can be expressed as

$$\rho_f(E) = \sum_n \int \mathrm{d}|g|\, \mathrm{D}(|g|)\mathrm{P}_n(|g|)\rho_i\big(E_{\mathrm{e},0} + n\hbar\omega\big). \tag{34}$$

and the coupling parameter can be expressed as

$$|g|_{\mathrm{ave}} = \int |g| \cdot \mathrm{D}(|g|)\mathrm{d}|g|. \tag{35}$$

Supplementary Figures

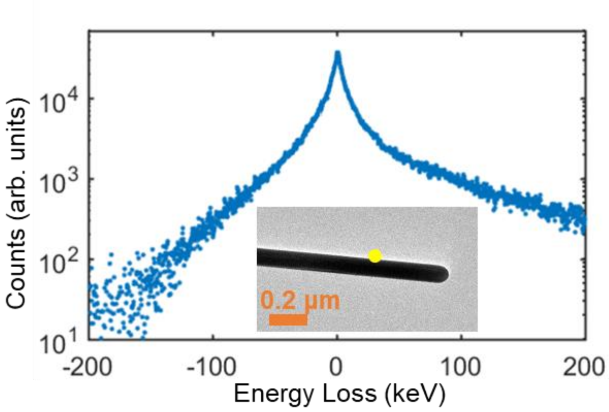

Fig. S1 | The electron spectrum from a single nanowire pumped by a laser with fluence about 35 mJ/cm^2. Electron beam parameters and other laser beam parameters are the same with the parameters demonstrated in the main text. For a single silver nanowire with diameter of about 100 nm, the highest electron energy-gain can be up to 150 eV. The inset shows the bright-field image of the nanowire. The yellow dot represents the detection position.

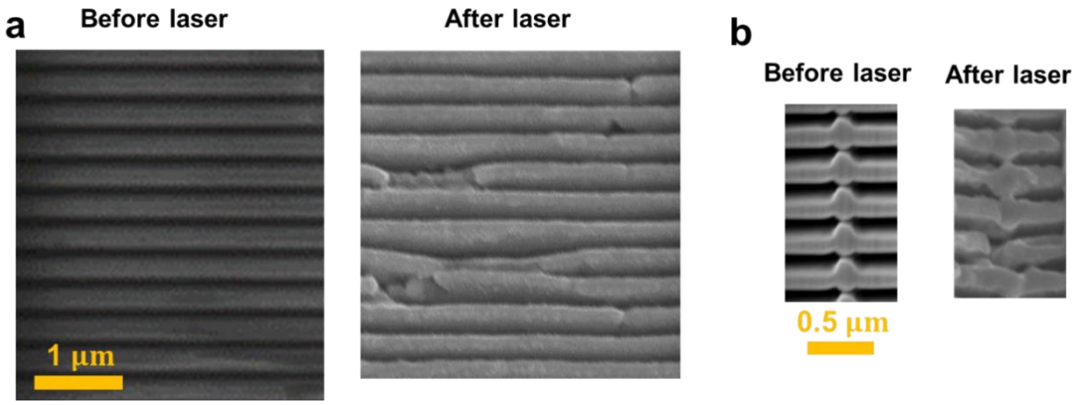

Fig. S2 | MLA images before and after laser irradiation with a fluence of 51 mJ/cm^2. Electron beam parameters and other laser beam parameters are the same with the parameters demonstrated in the main text. The corresponding laser intensity is 2.55×10^{11} W/cm^2, driving optical field is 1.38 GV/m. **a**, MLA with simple grating structures. **b**, MLA with bowtie structures.

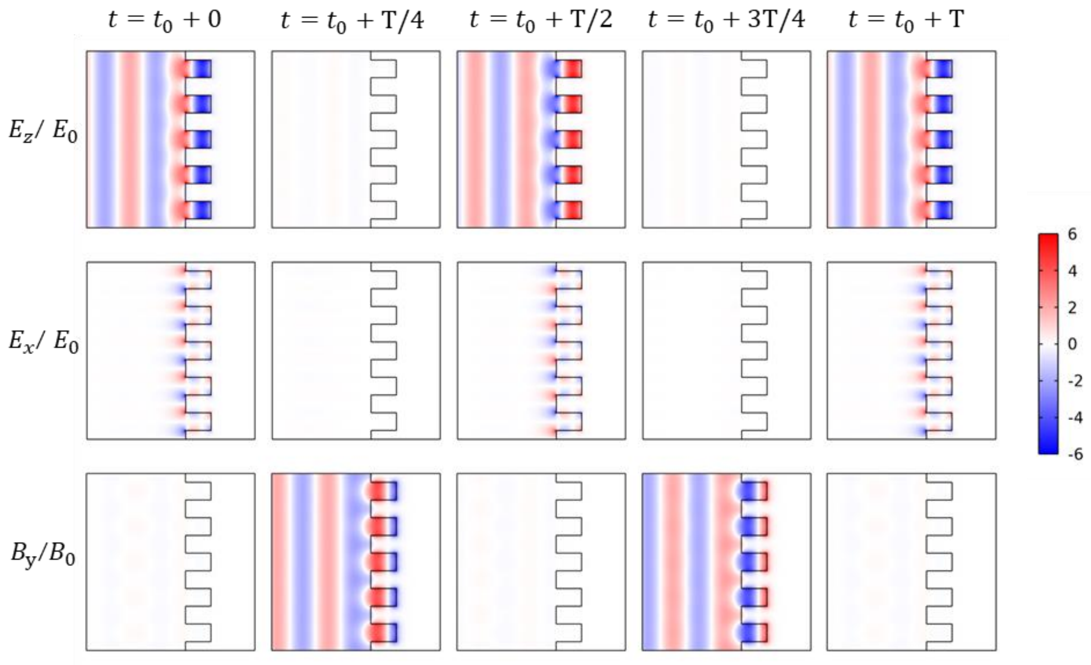

Fig. S3 | Simulated results of time-dependent electric and magnetic field distributions on MLA.

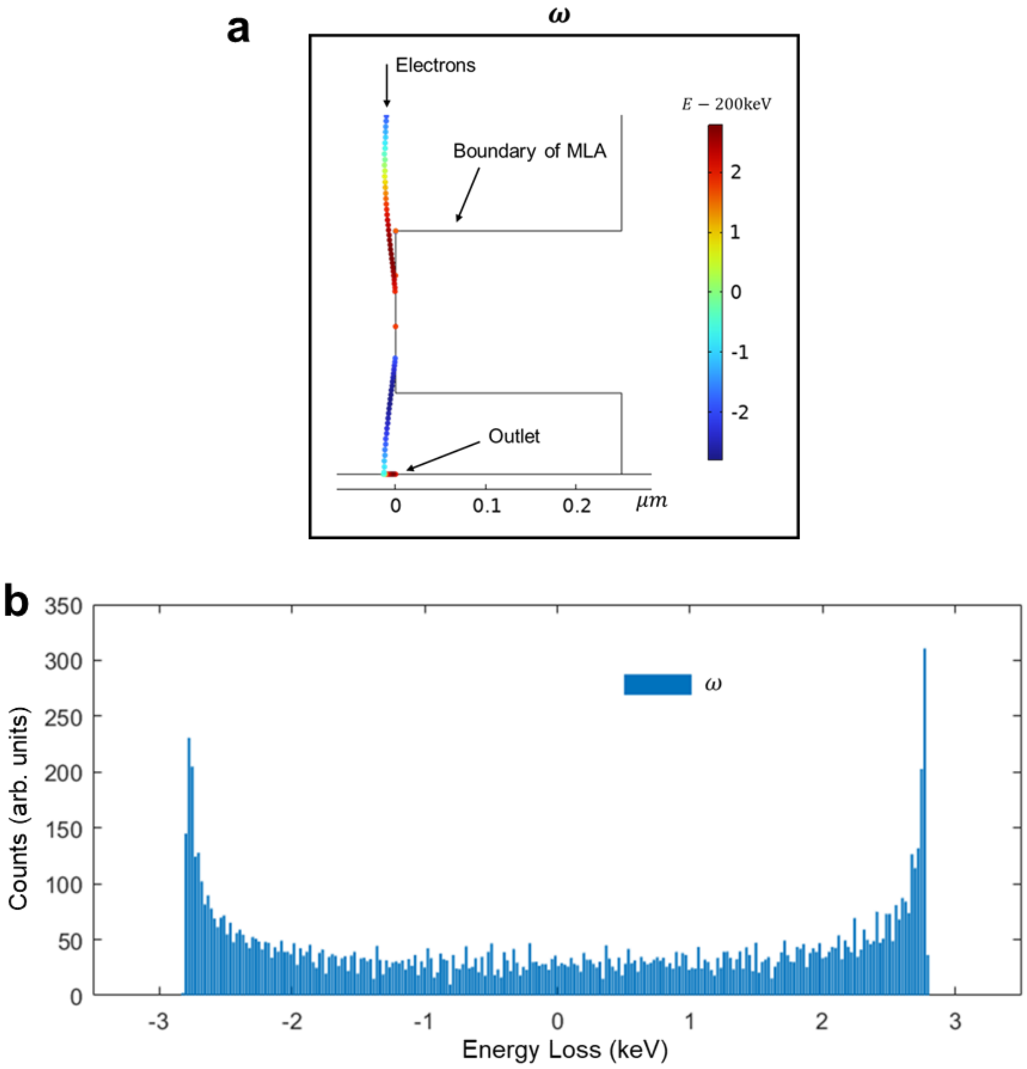

Fig. S4 | All electrons have an impact factor of 5 nm, the temporal width of laser pulse is much large than electron pulse, a close-up of electrons pass last tooth of MLA is shown in **a**, where some electrons are hit on the boundary of MLA and doesn't reach to outlet. The final electron energy spectrum shown in **b** is symmetric, which indicates that partial electrons hitting nanostructure cannot result in spectrum asymmetric.

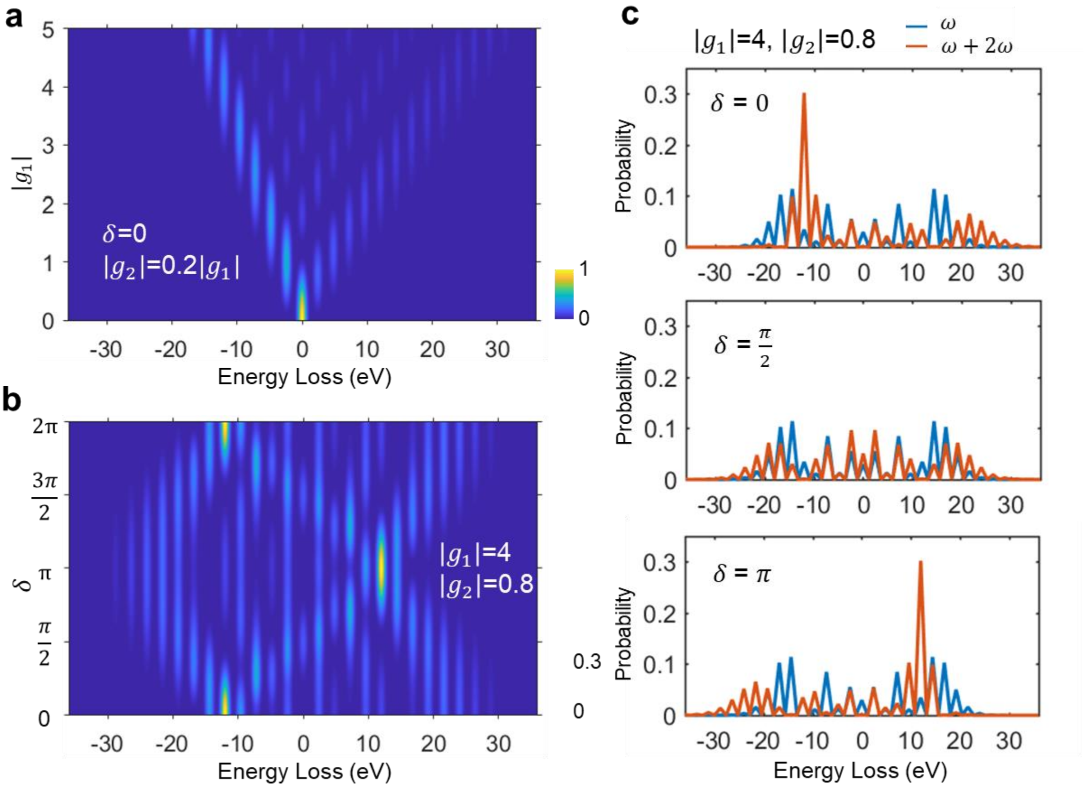

Fig. S5 | A typical Electron density distribution while both fundamental and SHG fields exist. **a**, Probabilities of electron in each energy state varied with near field intensity. **b**, Probabilities of electron in each energy state varied with relative phase. **c,** Three sections of **b** and the corresponding electron spectrum with only fundamental field.

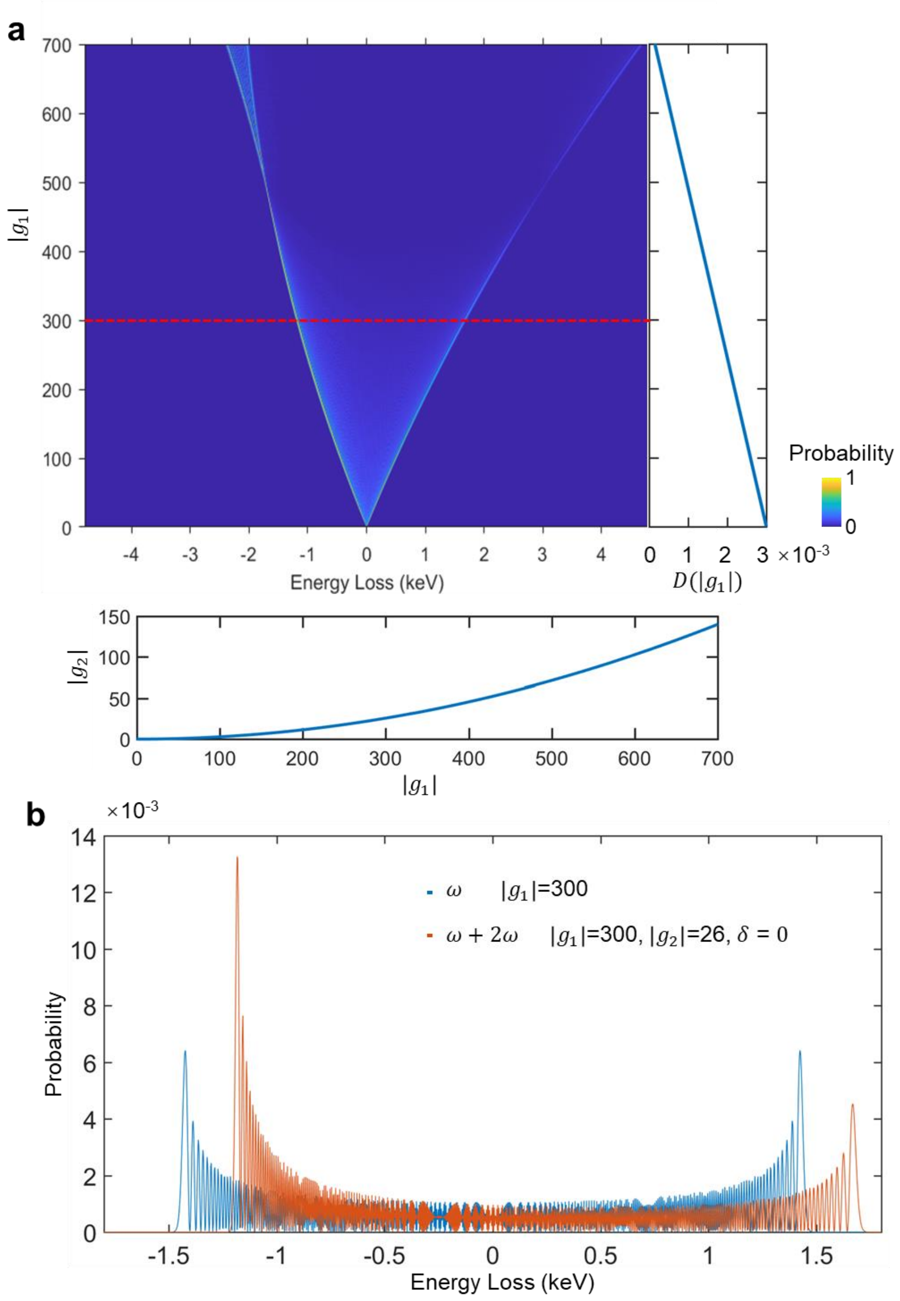

Fig. S6 | a, Near-field intensity-dependent probability distribution of electron in each photon order. Fundamental near field is coexistence with SHG. Each electron spectrum had normalized to its maximum. Assuming $|g_1|$ and $|g_2|$ have quadratic relation, which is shown in below. Because electrons experience different near-field intensity (different $|g_1|$), we assume the probabilities of electron in different $|g_1|$ could be expressed as a linear relation, as shown in the right. The definition of $D(|g|)$ see SI 2.3. **b**, The electron spectrum indicated by red dash line in **a**, as well as the spectrum for only fundamental field.

Table S1 | A comparison of MLA with DLA. In our experiments, interaction length is 7.16 μm, and fluence is 25.5 mJ/cm^2 (corresponding to intensity of 1.27×10^{11} W/cm^2). Because the laser size (diameter ~50 μm) is much larger than the interaction area, the acceleration gradient formula provided in reference[8] doesn't apply.

Initial electrons energy	Material	Maximum energy gain (keV)	Incident electric field E_0 (GV/m)	Acceleration gradient G (MV/m)	Efficiency $f = G/E_0$	Works
60 MeV	SiO_2	115	3.2	309.8	0.097	1
8 MeV	SiO_2	18	6	850	0.14	2
96.3 keV	Si	1.95	0.904	376	0.42	3
83.4 keV	Si	0.915	0.335	30.5	0.091	4
200 keV	Ag	2.4	0.978	335	0.34	Our work

Features: 1. Proof-of-principle experiment[9]. 2. Highest record for relativistic electrons[10]. 3. Highest record for subrelativistic electrons[11]. 4. On-Chip Integrated waveguide-integrated[12].

Supplementary References

1 Plettner, T., Lu, P. P. & Byer, R. L. Proposed few-optical cycle laser-driven particle accelerator structure. *Physical Review Special Topics - Accelerators and Beams* **9**, 111301, doi:10.1103/PhysRevSTAB.9.111301 (2006).

2 Bar-Lev, D. & Scheuer, J. Plasmonic metasurface for efficient ultrashort pulse laser-driven particle acceleration. *Physical Review Special Topics - Accelerators and Beams* **17**, 121302, doi:10.1103/PhysRevSTAB.17.121302 (2014).

3 Garcia de Abajo, F. J., Asenjo-Garcia, A. & Kociak, M. Multiphoton absorption and emission by interaction of swift electrons with evanescent light fields. *Nano Lett* **10**, 1859-1863, doi:10.1021/nl100613s (2010).

4 Park, S. T., Lin, M. & Zewail, A. H. Photon-induced near-field electron microscopy (PINEM): theoretical and experimental. *New Journal of Physics* **12**, doi:10.1088/1367-2630/12/12/123028 (2010).

5 Di Giulio, V., Kociak, M. & de Abajo, F. J. G. Probing quantum optical excitations with fast electrons. *Optica* **6**, 1524-1534, doi:10.1364/OPTICA.6.001524 (2019).

6 Kfir, O. Entanglements of Electrons and Cavity Photons in the Strong-Coupling Regime. *Physical Review Letters* **123**, 103602, doi:10.1103/PhysRevLett.123.103602 (2019).

7 Dahan, R. *et al.* Resonant phase-matching between a light wave and a free-electron wavefunction. *Nature Physics* **16**, 1123-1131, doi:10.1038/s41567-020-01042-w (2020).

8 Breuer, J., Graf, R., Apolonski, A. & Hommelhoff, P. Dielectric laser acceleration of nonrelativistic electrons at a single fused silica grating structure: Experimental part. *Physical Review Special Topics - Accelerators and Beams* **17**, 021301, doi:10.1103/PhysRevSTAB.17.021301 (2014).

9 Peralta, E. A. *et al.* Demonstration of electron acceleration in a laser-driven dielectric microstructure. *Nature* **503**, 91-94, doi:10.1038/nature12664 (2013).

10 Cesar, D. *et al.* High-field nonlinear optical response and phase control in a dielectric laser accelerator. *Communications Physics* **1**, 46, doi:10.1038/s42005-018-0047-y (2018).

11 Leedle, K. J. *et al.* Dielectric laser acceleration of sub-100 keV electrons with silicon dual-pillar grating structures. *Opt. Lett.* **40**, 4344-4347, doi:10.1364/OL.40.004344 (2015).

12 Sapra, N. V. *et al.* On-chip integrated laser-driven particle accelerator. *science* **367**, 79-83, doi:10.1126/SCIENCE.AAY5734 (2020).
